# Phase II Trial of Sipuleucel-T and Stereotactic Ablative Body Radiation for Patients with Metastatic Castrate-Resistant Prostate Cancer

**DOI:** 10.3390/biomedicines10061419

**Published:** 2022-06-15

**Authors:** Raquibul Hannan, Michael J. Dohopolski, Laurentiu M. Pop, Samantha Mannala, Lori Watumull, Dana Mathews, Ang Gao, Aurelie Garant, Yull E. Arriaga, Isaac Bowman, Jin-Sung Chung, Jing Wang, Kiyoshi Ariizumi, Chul Ahn, Robert Timmerman, Kevin Courtney

**Affiliations:** 1 Department of Radiation Oncology, UT Southwestern Medical Center, Dallas, TX 75390, USA; michael.dohopolski@utsouthwestern.edu (M.J.D.); laurentiu.pop@utsouthwestern.edu (L.M.P.); samantha.mannala@utsouthwestern.edu (S.M.); aurelie.garant@utsouthwestern.edu (A.G.); jing.wang@utsouthwestern.edu (J.W.); robert.timmerman@utsouthwestern.edu (R.T.); 2 Department of Radiology, UT Southwestern Medical Center, Dallas, TX 75390, USA; lori.watumull@utsouthwestern.edu (L.W.); dana.mathews@utsouthwestern.edu (D.M.); 3 Department of Population and Data Sciences, UT Southwestern Medical Center, Dallas, TX 75390, USA; ang.gao@utsouthwestern.edu (A.G.); chul.ahn@utsouthwestern.edu (C.A.); 4 Department of Medical Oncology, UT Southwestern Medical Center, Dallas, TX 75390, USA; yull.edwin.arriaga@ibm.com (Y.E.A.); isaac.bowman@utsouthwestern.edu (I.B.); kevin.courtney@utsouthwestern.edu (K.C.); 5 Department of Dermatology, UT Southwestern Medical Center, Dallas, TX 75390, USA; jin-sung.chung@utsouthwestern.edu (J.-S.C.); kiyoshi.ariizumi@utsouthwestern.edu (K.A.); 6 Dermatology Section (Medical Service), North Texas Veterans Affairs, Dallas, TX 75390, USA

**Keywords:** metastatic castrate-resistant prostate cancer, stereotactic ablative radiotherapy, sipuleucel-T, immunotherapy, clinical trial

## Abstract

(1) We hypothesized that adding concurrent stereotactic ablative radiotherapy (SAbR) would increase the time to progression in patients with metastatic castrate-resistant prostate cancer (mCRPCA) treated with sipuleucel-T. (2) Patients with a history of prostate cancer (PC), radiographic evidence of metastatic disease, and rising prostate-specific antigen (PSA) > 0.2 ng/dL on castrate testosterone levels were enrolled in this single-arm phase II clinical trial and treated with sipuleucel-T and SAbR. The primary endpoint was time to progression (TTP). Cellular and humoral responses were measured using ELISpot and Luminex multiplex assays, respectively. (3) Twenty patients with mCRPC were enrolled and treated with SAbR to 1–3 sites. Treatment was well tolerated with 51, 8, and 4 treatment-related grade 1, 2, and 3 toxicities, respectively, and no grade 4 or 5 adverse events. At a median follow-up of 15.5 months, the median TTP was 11.2 weeks (95% CI; 6.8–14.0 weeks). Median OS was 76.8 weeks (95% CI; 41.6–130.8 weeks). This regimen induced both humoral and cellular immune responses. Baseline M-MDSC levels were elevated in mCRPC patients compared to healthy donors (*p* = 0.004) and a decline in M-MDSC was associated with biochemical response (*p* = 0.044). Responders had lower baseline uric acid levels (*p* = 0.05). No clear correlation with radiographic response was observed. (4) While the regimen was safe, the PC-antigen-specific immune response induced by SAbR did not yield a synergistic clinical benefit for patients treated with sipuleucel-T compared to the historically reported outcomes.

## 1. Introduction

About 9600 men with prostate cancer (PC), the most common malignancy in men, will present with metastatic disease (mPC) at diagnosis this year, and a good proportion that initially present with localized disease will eventually develop mPC, where the 5-year relative survival is only 30% [1]. Standard of care treatment approaches for mPC include castration therapy to suppress androgen production (androgen deprivation therapy [ADT]), with or without additional agents that have demonstrated life-prolonging benefits: docetaxel chemotherapy and oral androgen signaling inhibitors abiraterone, enzalutamide, and apalutamide [2,3,4,5]. However, many patients ultimately develop castration-resistant PC (CRPC), for which the median overall survival (OS) historically ranges between 1 and 2 years [6,7]. A more complete summary of treatments for metastatic CRPC (mCRPC) includes docetaxel, cabazitaxel, abiraterone, enzalutamide, radium-223, lutetium-177-PSMA-617, and sipuleucel-T, all of which have been associated with improvements in OS [8,9,10,11].

Immunotherapies seek to stimulate the immune system to recognize and kill cancer cells. Sipuleucel-T, the only approved immunotherapy for PC, is a therapeutic cancer vaccine composed of autologous antigen-presenting cells (APCs) extracted via leukapheresis that is activated ex vivo with a fusion protein (PA2024), which contains antigen prostatic acid phosphatase (PAP) and granulocyte-macrophage colony-stimulating factor (GM-CSF), a cytokine that stimulates APC maturation. Sipuleucel-T has been shown in multiple clinical trials to increase survival in patients with CRPC [12,13]. However, median survival is typically increased by only 4–7 months. Men with mPC are thought to have immunosuppressive tumor microenvironments that limit immune cell circulation, expansion, and infiltration and, thus, likely reduce the effectiveness of immune-based therapies such as sipuleucel-T [14]. Highly focused, high-dose radiation therapy (stereotactic ablative radiotherapy; SAbR) has been shown in pre-clinical and clinical settings to have immunogenic properties, including improved antigen presentation by APCs, T-cell migration and priming, and modulation of regulatory T cells [15,16,17,18,19]. The combination of immune-based systemic therapy and SAbR has shown encouraging results in managing metastatic melanoma [20]. SAbR has highly cytoreductive properties, which are critical for decreasing the overall disease burden. Huang et al. showed that disease burden in patients with metastatic melanoma was related to T-cell exhaustion and poor progression-free survival (PFS) [21]. While decreasing the overall tumor burden can directly lead to survival benefit, it is also hypothesized to reduce immunosuppression and thereby contribute to T-cell reinvigoration and synergy with immunotherapy, thus improving clinical outcomes for patients receiving immunotherapy [21,22].

Patients with metastatic PC can present with bulky disease and innumerable sites of micrometastatic disease. Systemic therapy is effective in treating sites of micrometastatic disease but likely less effective in treating bulky disease. SAbR is a cytoreductive modality that can safely target bulky disease sites and provide immunostimulatory effects. Therefore, we report the results of a prospective single-arm, open-label, institutional review board-approved and registered (NCT01818986) phase II trial evaluating the concurrent use of sipuleucel-T and SAbR to bulky sites of metastasis for men with mCRPC. We hypothesized that sipuleucel-T combined with SAbR would increase the time to progression compared to the historically reported registration trial of sipuleucel-T alone [12].

## 2. Methods

### 2.1. Patients

After providing written informed consent, patients were enrolled in the trial within our single institution if they had mCRPC with identifiable metastases by standard imaging (CT, MRI, bone scan), increasing prostate-specific antigen (PSA) beyond 0.2 ng/mL confirmed with a second measurement in the setting of testosterone < 50 ng/dL, Eastern Cooperative Oncology Group (ECOG) performance status of 0–1, age ≥ 18 years, and a life expectancy of ≥6 months. They must have been receiving ADT or anti-androgen therapy with a serum testosterone level < 50 ng/dL and had radiographic evidence of metastatic disease documented on bone scan, CT scan, MRI, or PET scan.

Patients were excluded if they had initiated or discontinued bisphosphonates within 14 days before enrollment, had received chemotherapy or systemic corticosteroids (unless used in combination with abiraterone) within 14 days of enrollment, had metastatic disease exclusively present within previously irradiated fields, or had spinal cord compression, Paget’s disease of the bone, brain metastases, malignant pleural effusions, malignant ascites, or a history of positive serology tests for human immunodeficiency virus (HIV) 1 and 2, human T cell lymphotropic virus (HTLV)-1, or Hepatitis B or C.

### 2.2. Treatment

Sipuleucel-T can be administered as any line of therapy for the management of mCRPC (ie 1st line, 2nd line, 3rd line, etc.). Three cycles of sipuleucel-T were administered every two weeks following the standard of care treatment protocol [12]. SAbR was delivered during the third or fourth week but prior to the last leukapheresis. Up to six sites were allowed to be treated with SAbR. One (total dose 21–27 Gy) or three (total dose 26.5–33 Gy) fractions were allowed. Preference was given to the largest disease sites feasible for treatment, bulky progressive sites, symptomatic sites, and sites where palliative and preventative indications existed. All SAbR plans were optimized to ensure adequate target volume coverage by the prescription dose. Normal tissue constraints were used as previously described [23]. Breath-hold, respiratory gating, or abdominal compression techniques and image-guided radiation were used as needed at the discretion of the treating radiation oncologist.

### 2.3. Target Lesion Criteria

All measurable lesions, up to five lesions total (and a maximum of two lesions per organ), representative of all involved organs, should be identified as target lesions. All other lesions (or sites of disease), including any measurable lesions over and above the five target lesions, should be identified as non-target lesions. Radiated lesions were excluded in RECIST measurements.

### 2.4. Adverse Events (AEs) and Follow-Up

Physical exams, laboratory analysis, and imaging studies were performed every 8–12 weeks after treatment until radiographic progression, at which point patients were followed for survival. Standard imaging with bone and CT scans was obtained, and laboratory specimens, including for PSA, were performed at each follow-up visit. Adverse events (AEs) were graded based on the Common Terminology Criteria for AEs, version 4.0 (CTCAE 4.0). Toxicity attribution was reviewed monthly by a panel of physicians. Immunologic correlates obtained from serum blood samples were obtained before the combination therapy, after SAbR, and 6, 12, and 24 weeks following the completion of sipuleucel-T. The final data update/review was performed 11 November 2019.

### 2.5. Cellular Response Measurement by (Interferon) IFNg ELISpot

Peripheral cellular responses specific to the PA2024 and PAP antigens were assessed using IFNg enzyme-linked immune absorbent spot (ELISpot) assays as previously described [24]. Briefly, thawed and re-cultured cryopreserved peripheral blood mononuclear cells (PBMCs) were plated on polyvinylidene fluoride ELISpot plates (Millipore, Billerica, MA, USA) at a concentration of 0.3 × 10^6^ cells/well/200 mL media with or without PA2404 or PAP proteins. Murine anti-human IFNg monoclonal antibodies (clones 1-D1K and 7-B6-1, respectively, MABTECH, Upsala, Sweden) were used for IFNg capture and detection, followed by streptavidin-alkaline phosphatase and its substrate (BCIP/NBT), per the manufacturers’ instructions.

### 2.6. Humoral Response Measurement by Luminex Multiplex Assay

Humoral immune responses against PA2024, PAP, and non-targeted tumor antigens identified in the IMPACT clinical trial were measured as previously described [24]. These included prostate-specific antigens, embryonic stem-cell–expressed Ras (ERAS), Kirsten rat sarcoma viral oncogene (KRAS), kallikrein-related peptidase 2 (KLK2), galectin-3 (LGASL3), galectin-8 (LGALS8), and tetanus (as a control for the assay). Titers of IgG antibodies specific against the aforementioned antigens were measured using Luminex^®^ xMAP^®^ (Luminex Corporation, Austin, TX, USA), a multiplexed, antigen-coupled, spectrally distinguishable, fluorescent bead method. Normalized signal (fluorescence) intensities from Luminex xMAP were log2-transformed before final analysis.

### 2.7. Investigation of Myeloid-Derived Suppressor Cells (MDSCs) by Fluorescence-Activated Cell Sorting (FACS)

PBMCs previously isolated by Ficoll-Paque gradient assay [25] and cryopreserved in liquid nitrogen were thawed and stained with the following fluorescence-labeled anti-human monoclonal antibodies (MAbs): FITC-anti-CD14 (Invitrogen. Waltham, MA, USA), PE-anti-CD33 (eBioscience, Carlsbad, CA, USA), PerCP/Cy5.5-anti-CD235ab (BioLegend, San Diego, CA, USA), APC-anti-HLA-DR (Invitrogen), APC-Cy7-anti-CD11b (Invitrogen), Brilliant Violet 421-CD15 (BioLegend), and Brilliant Violet 510-Lineage Cocktail (BioLegend) and analyzed using DB FACSVerse (BD Bioscience). Staining with anti-CD235 MAb was used to exclude non-nucleated red blood cells. Live PBMCs were gated in size-scatter vs. forward-scatter plot. Monocytic-MDSC (M-MDSC) was identified by the phenotype of CD14^+^HLA-DR^low/−^; andearly-stage-MDSC (e-MDSC) was identified in Lineage^−^/HLA-DR^low/−^ fraction as CD11b^+^CD33^+^ phenotype. PMN-MDSC were not assessed due to the well-known sensitivity of this MDSC subpopulation in cryopreserved samples [26].

### 2.8. Statistical Analysis

The primary endpoint was time to progression (TTP). TTP was measured from initiation of therapy to disease progression, as described by the Prostate Cancer Clinical Trials Working Group 2 and the IMPACT trial [12,27]. Disease progression was defined as a >50% increase (in the sum of the products of diameters for index lesions) in measurable disease (RECIST 1.1), apparent worsening of non-measurable disease, or appearance of >1 new bone lesion observed via bone scan, confirmed by a repeat bone scan after ≥6 weeks. Two board-certified radiologists (LW, DM) completed the response assessments. The appearance of two new lesions on the first follow-up scan (6-week scan) required ≥2 additional new lesions in a repeat bone scan ≥ 6 weeks apart. The development of spinal cord compression, nerve root compression, or a pathologic fracture were also defined as progressive events.

The estimated sample size to observe an increase in TTP of ≥80% over the historical control of 14.6 weeks with sipuleucel-T alone, with a two-sided test significance level of 10% and 80% power, was 20, assuming an accrual period of 3 years and a follow-up period of 4 years.

Secondary endpoints were overall survival (OS), PC-specific survival (PCaSS), progression-free survival (PFS), biochemical progression-free survival (bPFS), and AEs. OS, PCaSS, PFS, and bPFS were measured from the initiation of combination therapy to their respective endpoints. OS was defined as the duration of time from the start of treatment to the time of death from any cause. PCaSS was defined as the percentage of patients who had not died from PC at the time of analysis. PFS was defined as the length of time from the start of treatment to disease progression or death from any cause. bPFS was defined as the time from the beginning of treatment to PSA disease progression. Biochemical progression was defined as an increase in PSA of >2 ng/mL from baseline and an increase of >25% from the baseline value and confirmed by a second measurement more than three weeks later. Post hoc analyses comparing the patient characteristics of patients who responded vs. those who did not respond to therapy can be found in Supplementary Table S1.

The immunologic endpoint was an amplification of immune response (compared to historically reported values for sipuleucel-T) [28]. There had to be a >100% increase in immune response, as measured by Sheikh et al. for patients treated in the IMPACT trial to reach the immunologic endpoint [24]. The changes in humoral and cellular responses were calculated using the unequal variance Welch t-test. A *p*-value less than 0.05 (*p* < 0.05) was considered statistically significant. The figures were developed, and analyses were performed using GraphPad Prism software version 9.0 for Windows (GraphPad Software, San Diego, CA, USA). Two-sample t-tests were conducted to examine if there were significant differences in baseline, follow-up, and changes in MDSC, titers of antibodies, and complete blood count with differential between clinical and PSA responders and non-responders. Two-sample t-tests were also used to investigate if there were significant differences in baseline MDSC between patients and healthy donors.

## 3. Results

From July 2013 to October 2018, twenty patients were enrolled after giving written informed consent. Patient demographics and clinical data are summarized in Table 1. The median follow-up was 15.5 months. The mean age at diagnosis was 63 years. These men all had adenocarcinoma histology (100%), with one patient having a cribriform variant, and most had grade group 4–5 disease (89%) and were stage IIIB-IVB (95%) at the time of their initial diagnoses. Fifty-five percent of these men had a high burden of metastatic disease, as defined in the CHAARTED trial, at enrollment [5]. The trial stopped enrolling patients after meeting its accrual goal of 20 patients.

All patients received the three planned cycles of sipuleucel-T. The median time from the first cycle to the third cycle of sipuleucel-T was 28 days (standard deviation 5.9 days). Five patients received SAbR before Cycle 2. Seventeen patients received SAbR treatment within one week of Cycle 2. Two of the other three patients received SAbR treatment 18 days before Cycle 2, and the other patient received SAbR treatment ten days before Cycle 2. All patients received SAbR between Cycles 1 and 3 as planned. SAbR targets are listed in Table 1. The most common site of SAbR was bone, followed by lymph nodes, then the prostate itself. Most patients received radiation to one site. The median number of systemic therapies received before enrollment was three (range 2–9); seven patients had ≥ five systemic therapies before enrollment.

The median dose and number of fractions were 2700 cGy and 3 fractions, respectively. Doses ranged from 2000–3000 cGy, and only 1 or 3 fractions were given. Single-fraction treatments all targeted bony lesions.

### 3.1. Outcome

**Figure 1:**
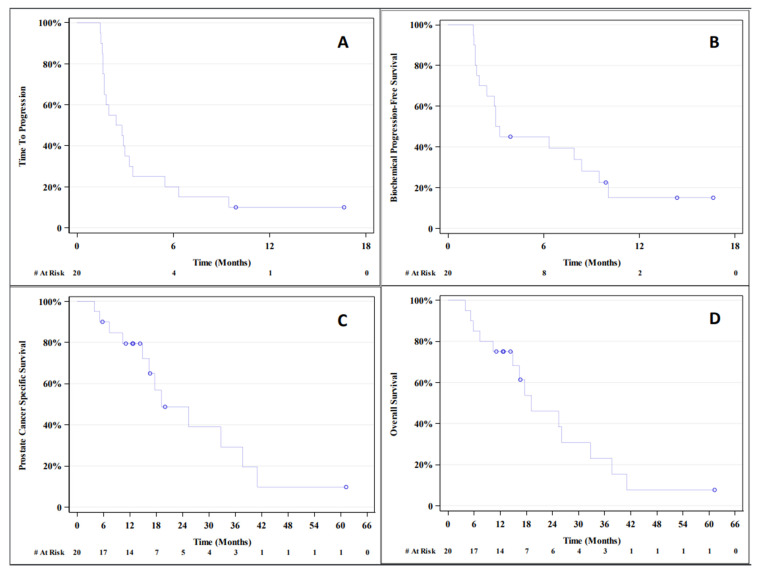
Kaplan-Meier plots for time to (**A**) progression (TTP), (**B**) biochemical progression-free survival (bPFS), (**C**) prostate cancer-specific survival (PCaSS), and (**D**) overall survival (OS).

The median TTP was 11.2 weeks (95% CI; 6.8–14.0 weeks), which was not numerically higher than that reported in the historical control from Kantoff et al. (14.6 weeks). The median PFS was 10.4 weeks (95% CI; 6.4–13.9 weeks). The median PCaSS was 77.6 weeks (95% CI; 59.6–150.8 weeks). The median OS was 76.8 weeks (95% CI; 41.6–130.8 weeks); the historic control was 112.1 weeks, as reported in Kantoff et al. The median bPFS was 12.5 weeks (95% CI; 4.8–33.5 weeks). The Kaplan-Meier plots for TTP, bPFS, PCaSS, and OS are shown in Figure 1.

**Figure 2:**
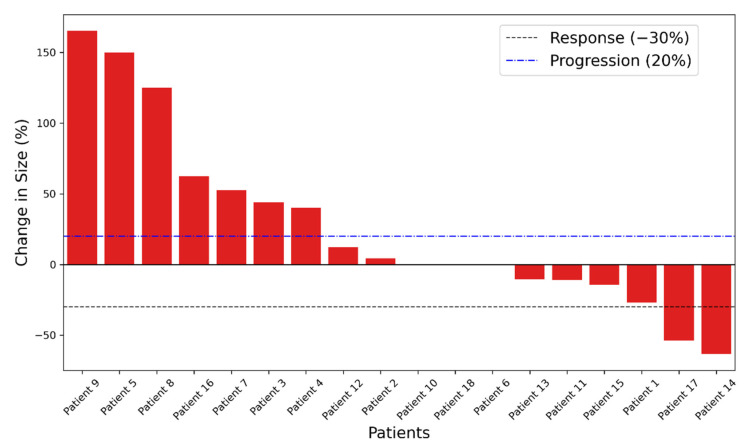
Waterfall plot displaying the change in the sum of the longest diameter compared to baseline measurements for patients receiving sipuleucel-T.

Eleven patients had stable or partial disease response as their best overall RECIST response, as seen in the waterfall plot in Figure 2.

### 3.2. Toxicity

A majority of patients experienced grade 1 or 2 AEs. Prominent grade 1 events included fatigue and nausea. Only four grade 3 AEs were observed (chills, fatigue, nausea, and vomiting). No grade 4+ events were observed. A detailed list of AEs is shown in Supplementary Table S2.

### 3.3. Immunologic Endpoint

**Figure 3:**
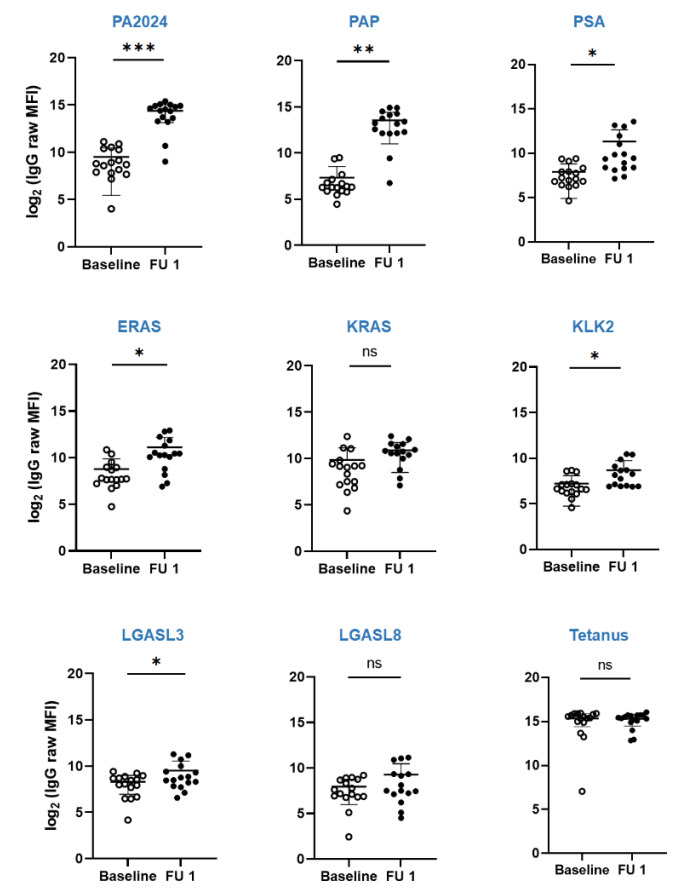
Dynamics of humoral response as measured via Luminex multiplex assay of sera titers of antibodies against PA2404, PAP, PSA, ERAS, KRAS, KLK2, LGASL3, LGASL8, and tetanus at baseline (open circle) and follow-up visit (closed circle) for patients enrolled in the trial. * *p* <0.05, ** *p* < 0.005, *** *p* < 0.0005, ns–not significant.

In 15 out of 16 patients, where sera were available for both pre- and post-treatment time points, the antibody titers against PA2024 and PAP significantly increased at follow-up visits, irrespective of clinical response (Figure 3). One patient (#12) who did not respond was completely immune “silent” (e.g., no changes when compared to baseline) for both humoral and cellular responses. Sera antibody titers against non-target antigens were also significantly increased for PSA, ERAS, KLK2, and LGASL3 (Figure 3), which suggests antigenic spread.

The patients with the best radiographic responses (#5, 8, and 9; Figure 2) had a moderate increase in the titers of post-treatment antibodies against PA2024 (23 to 82 folds) and PAP (29 to 393 folds). In contrast, the patients with the highest titers (11 to 2074 folds in #17 and 73 to 623 folds in #14) did not necessarily exhibit the best radiographic responses. Of note, patient 17, who had the most significant increment in the anti-PA2024 and PAP titers, also had a considerable increment in all antibodies against the tested antigens (including 55-fold against tetanus), which suggests an over-amplified non-specific humoral immune response.

**Figure 4:**
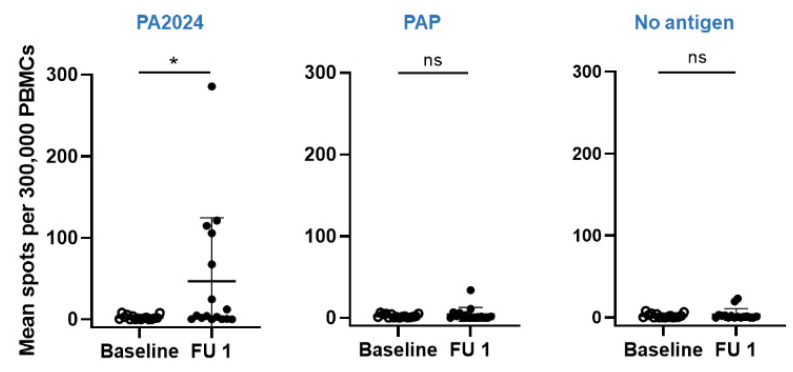
Dynamics of cellular response as measured by the number of IFNγ spots in the ELISpot assay against PA2404, PAP, and no antigen (control) at baseline (open circle) and follow-up visit (closed circle). * *p* < 0.05, ns–not significant.

The PA2024-specific cellular response was present in 50% (8 out of 16) of patients, but there was no correlation with disease response (Figure 4). However, the number of IFNg spots increased significantly in four patients with progressive disease (PD) and two patients without PD. Interestingly, patient 17, who had the exacerbated humoral response, had zero IFNg spots both at baseline and at follow-up visits, suggesting that in his predominating humoral response, the activation of specific anti-tumor CD8^+^ T cells were inhibited or diminished.

### 3.4. Responders vs. Non-Responders Characteristics

Patient characteristics were similar between responders vs. non-responders for both clinical/radiographic response and PSA biochemical response. The only noted difference was that the mean uric acid value at baseline was lower in the responder groups (Supplementary Table S1).

### 3.5. Dynamics of Circulating MDSCs in Responders vs. Non-Responders

We examined using flow cytometry the levels of the M-MDSC and e-MDSC populations in the cryopreserved PBMC at baseline and at the first follow-up visit from 2 to 4 months after the last infusion of sipuleucel-T. Flow cytometry gates used to identify both MDSCs sub-populations are shown in Supplementary Figure S1.

The baseline percentages of M-MDSC populations were much higher in all patients in this trial than healthy donors (9.73 ± 6.68 vs. 0.67 ± 0.31, *p* = 0.004) (Table 2A). There were also distinct differences in the percentages of M-MDSC at baseline among patients with or without radiographic response (12.06 ± 8.16 vs. 6.83 ± 3.14, *p* = 0.269) (Table 2B). After 2–4 months post-treatment, percentages of M-MDSCs decreased in radiographic partial responders (−2.56 ± 7.06%), whereas in patients with progressive disease, the percentages of M-MDSCs increased (+5.23 ± 3.49%) (*p* = 0.088) (Table 2B). Interestingly, a more significant association was found when the same parameters were analyzed by grouping the patients based on their PSA response. The M-MDSC at baseline among patients with or without a PSA response was 14.25 ± 7.54 vs. 6.12 ± 3.14, respectively (*p* = 0.063) (Table 2C). Post-treatment, the percentages of M-MDSCs declined in patients with a PSA response (−3.93 ± 7.28%), whereas those patients without a PSA response increased dramatically (+4.82 ± 3.16%) (*p* = 0.044) (Table 2C).

The investigation of e-MDSC revealed a trend towards higher e-MDSC percentages at baseline in all patients compared to healthy donors (*p* = 0.362) (Table 2A). While there were no observed differences at baseline between e-MDSCs from both the radiographic and biochemical responding groups, there was an observed trend for e-MDSCs to decrease slowly in responders but faster in non-responders, nearly reaching the healthy donor levels (Table 2B,C).

## 4. Discussion

In this prospective phase 2 clinical trial, we explored the efficacy of using sipuleucel-T in combination with SAbR to treat men with mCRPC. We hypothesized that this combination therapy could improve time to progression from the historical results reported in the IMPACT trial [12]. In our trial, the median TTP and OS were 10.4 weeks and 77.6 weeks (17.8 months), respectively. Additionally, eleven patients had stable or responsive disease at the time of analysis. TTP and OS were numerically lower than reported in the IMPACT trial. The lack of improvement in TTP and OS in patients enrolled in this trial is likely explained by (1) a higher burden of disease and (2) progression through many more lines of therapy than patients in the IMPACT trial. Patients in our trial had higher PSA values at enrollment (93 ng/mL vs. 51 ng/mL) and a higher primary Gleason score ≥ 4 (89% vs. 58%), both of which correlate with outcomes such as poor OS and PFS [29,30]. Additionally, our patients received an average of four systemic therapies before enrollment, including abiraterone, enzalutamide, and nilutamide; these therapies were not approved at the time of IMPACT trial. Therefore, patients in our trial had more therapy-resistant and advanced disease, both of which compromise the immune system.

This trial used IMPACT as the historical control; however, other smaller randomized trials show similar TTP and OS to IMPACT. D9901 and D9902A were two randomized, placebo-controlled, double-blinded studies with identical original designs that evaluated the safety and efficacy of sipuleucel-T as a treatment for men with metastatic PC [13,31]. Higano et al. performed a combined analysis (147 patients who received sipuleucel-T) of these studies, whose patients had baseline PSA and a Gleason score (50.7 ng/mL and 64.4% of patients with Gleason sum ≤ 7) similar to those in the IMPACT trial [31]. Median TTP and median OS in this cohort of patients were 11.1 weeks and 23.2 months, respectively. A more recent phase II study evaluated sipuleucel-T immunotherapy preceded by palliative radiation therapy (30 Gy in 10 fractions) and sipuleucel-T alone in a similar patient cohort (mCRPC with median Gleason score 7–8 but median baseline PSA of 13–20 ng/mL). There was a trend toward significantly better PFS, a secondary endpoint, in the patients who received radiation (3.65 months vs. 2.46 months, *p* = 0.06) [32]. They similarly noted that a moderate portion (32%) of patients had at least stable disease after therapy; in our cohort, 55% of patients had stable or responsive disease.

The side effects of sipuleucel-T are typically well-tolerated and manageable, with the majority being headaches, fevers, and chills [32]. There were eight grade 2 AEs and four grade 3 AEs in this trial. The grade 3 events were chills, fatigue, nausea, and vomiting. There were no grade 4 or greater AEs. Altogether, this suggests that the combination of SAbR with sipuleucel-T was well tolerated in this trial. These data add to the growing literature on the safety of concurrent SAbR and immunotherapy [33,34].

It has been previously demonstrated and was confirmed in this study that sipuleucel-T induces a robust humoral response against both targeted and non-targeted tumor antigens in most immunized patients and a moderate cellular immune response in nearly half of the immunized patients [28,35]. However, without a proper control arm in this trial, it was impossible to directly compare immunologic assay results to quantitate SAbR’s contribution to the induction of humoral and cellular immune responses. Furthermore, the study was not adequately powered to detect immune response, which was a secondary endpoint.

Immune responses in the patients in this trial did not correlate with their clinical outcomes, which contradicts some of the previously published results [28,35]. The generation of a specific peptide-driven humoral response was demonstrated by the increased titers of antibodies against PA2024 and PAP after sipuleucel-T treatment. These titers reflect the induction of an antibody that reacts to a particular epitope. It is also possible that, despite the generation of high levels of antigen-specific antibodies, no PC cells were killed upon antibody–antigen interaction due to the loss of the PAP epitope(s) recognized by anti-PAP antibodies or the alterations/loss of downstream functional kinase activity in prostate cells. As previously discussed, a combination of more therapy-resistant advanced disease and a compromised immune system may have contributed to these results.

A compromised immune system in these patients was confirmed with the significantly higher levels of M-MDSCs in patients enrolled in this trial compared to healthy donors (*p* = 0.004). Increased activity of MDSCs is a determinant feature of immune-exclusive barriers in PC [36]. Multiple studies have demonstrated that the M-MDSC are the primary circulating MDSC sub-population in patients with mCRPC [36,37,38]. The results from MDSC investigation in this study strongly suggest that patients who respond (either radiographically or biochemically) have a significant reduction in circulating M-MDSCs at 2–4 months post treatment. Interestingly, the baseline levels of M-MDSC did not predict the response to therapy. Unexpectedly, the patients who had higher M-MDSCs levels at baseline were the patients with better clinical/PSA responses (Table 2). This suggests that the regimen (SAbR + Sipuleucel-T) may have an interaction with the MDSCs and may be able to suppress or inhibit, or at the least overcome, the immunosuppressive effects of the MDSCs in inducing a response. Interestingly, we found a significant association between declining M-MDSCs and both radiographic and biochemical response. Although associations do not mean causation, these trends generate the hypothesis that this regimen can lead to a decline in M-MDSCs, which may portend to a more favorable radiographic and biochemical response. A less likely explanation could be that a decline in overall tumor burden by this regimen, perhaps secondary to tumor debulking by SAbR, led to a decline in MDSCs.

The contribution of diminished M-MDSC-driven immunosuppression is underlined by the observed trend in increments of titers against PAP and PA2024 in both clinical and PSA-based responders (Supplementary Table S3). Despite not reaching significance, the higher IgG titers may explain the benefit observed in this pool of patients.

The paucity of research related to the dynamics of e-MDSC sub-population shows no difference in PBMC between PC patients and healthy donors [37]. Recently, e-MDSCs have also been characterized as immunosuppressive cells in patients with ovarian cancer; however, their functions have not yet been deciphered [39]. Our study shows a higher percentage of circulating e-MDSC in PC patients compared to healthy donors, but, interestingly, the numbers tend to normalize only in patients that did not respond to the regimen (*p* = 0.065). Nevertheless, e-MDSC requires further investigation in patients with cancer to define its roles in tumor immunity and as a potential biomarker.

Of note, on post hoc analyses, the mean uric acid levels in responders were significantly lower than in non-responders. It is likely that the increased uric acid levels are the result of increased cancer cell turnover. Increased uric acid levels have been shown in the literature to be a poor prognostic marker for advanced gastric cancer patients [40]. While uric acid acts as an anti-oxidant via scavenging free radicals, abnormal plasma levels trigger inflammation and oxidative stress [41,42]. There is some evidence to support that hyperuricemia leads to dysregulated T cell proliferation and can affect the response of cancer to immunotherapy [41,42]. Therefore, it remains to be evaluated if higher levels of plasma uric acid played any causative role in patients failing to respond to this regimen.

This trial has several limitations, the primary one being the small sample size. The study enrolled the required number of patients to assess the primary outcome as detailed above; however, the secondary analyses all suffered from the small sample size. Additionally, our median follow-up time was shorter than IMPACT’s (15.5 vs. 34.1 months). Another limitation of our study is that we did not have a randomized arm for comparison, so we used the IMPACT trial results as a historical control instead. As was detailed earlier, there are notable differences not only in the study populations but also in the available and approved systemic therapies. Finally, correlative immune studies were limited by the small sample size, the lack of a control arm, and the number of time points when blood was collected, and the availability of fresh PBMCs.

PC is considered an immune-suppressive cancer, as immunotherapies, in general, have failed to show notable responses [43,44,45]. In contrast to mCRPC, high-risk localized PC would be a better setting to evaluate the synergy between SAbR and immunotherapy for multiple reasons: (1) radiation is already a standard of care, (2) the PC is not sufficiently advanced to compromise the host’s immune system, and (3) more than 50% of high-risk PC patients relapse distantly, which can be used as a primary endpoint. Furthermore, the STAMPEDE trial has shown a benefit for treating the primary site in the setting of low burden metastatic PC, which is another context in which immunotherapy could be introduced [46]. Recently, the long-term results from CA184-043 (with a median follow-up of 38.7 months) showed an OS benefit (3-yr OS of 15.3% vs. 7.9%) for patients who received radiation therapy and ipilimumab vs. patients who received radiation alone. These data illustrate that certain immunotherapies in combination with radiation could benefit patients. However, this needs to be assessed in a larger randomized phase III trial with prolonged follow-up [45,47].

## 5. Conclusions

We evaluated the efficacy of concurrent sipuleucel-T with SAbR to treat men with mCRPC. We found that the combination of sipuleucel-T and SAbR did not increase TTP or OS compared to the original IMPACT trial. Nonetheless, this regimen was well tolerated. Furthermore, the increased cellular and humoral responses in patients with mCRPC immunized with sipuleucel-T and SAbR did not correlate with the observed clinical responses. Interestingly, an interaction of this regimen with M-MDSCs were identified, and an association was found between the responders to this regimen and a decline in M-MDSCs. Further studies, perhaps in the earlier stages of prostate cancer where a more responsive immune environment is expected, and in combination with different immunotherapies and radiation regimens, are required to optimally harness the immunogenic potential of SAbR for the treatment of prostate cancer.

## Figures and Tables

**Table 1:** Patient characteristics.

**Variables**	**Mean ± STD**
**Age at Diagnosis (years)**	63 ± 9
**Age at Enrollment (years)**	69 ± 8
**Prior systemic therapy**	4 ± 2
**PSA value at Enrollment (ng/dL)**	93 ± 270
**PSA < 2**	N = 1
**PSA ≥ 2–10**	N = 10
**PSA ≥ 10**	N = 9
**Testosterone level (ng/dL)**	7 ± 5
**LDH (U/L)**	196 ± 44
**CRP (mg/L)**	7.9 ± 8.4
**Beta2 (mcg/mL)**	2.5 ± 0.8
**Uric Acid (mg/dL)**	5.3 ± 1.7
**WBC (×10^9^/L)**	6.0 ± 1.5
**Neutrophils (×10^9^/L)**	3.9 ± 1.4
**Lymphocytes (×10^9^/L)**	1.3 ± 0.5
**Monocytes (×10^9^/L)**	0.5 ± 0.2
**Eosinophils (×10^9^/L)**	0.1 ± 0.1
**Variables**	**# (%)**
**Race**	
White, not Hispanic	14 (70%)
Black, not Hispanic	4 (20%)
Hispanic	1 (5%)
Asian	1 (5%)
**ECOG**	
0	12 (60%)
1	8 (40%)
**Grade Group**	
2–3	1 (5%)
4	4 (22%)
5	12 (67%)
**Original Primary Gleason Score**	
5	7 (35%)
4	10 (50%)
2–3	1 (5%)
**Original Secondary Gleason Score**	
5	8 (40%)
4	7 (35%)
3	2 (10%)
**Stage at Diagnosis**	
IIC	1 (5%)
IIIB	4 (21%)
IIIC	5 (26%)
IVB	9 (47%)
**High Burden Metastatic Disease**	
Yes	11 (55 %)
No	9 (45%)
**Histology**	
Adenocarcinoma	20 (100%)
**Variables**	**Number**
**SAbR Sites**	
Vertebral body	10
Bony pelvis	3
Non-pelvic/non-vertebral bony metastases	2
Pelvic lymph nodes	3
Para-aortic lymph nodes	1
Supraclavicular lymph nodes	1
Prostate	4
**Treatment Sites per Patient**	
1	11
2	5
3	1
**Dose/Fraction**	
20–21 Gy in 1 fraction	10
24, 27, 30 Gy in 3 fractions	14
**Systemic Therapy after sipuleucel-T**	
Radium	3
Olaparib	2
Mitoxantrone	1
Lupron	13
Enzalutamide	5
Docetaxel	11
Degarelix	1
Cyclophosphamide	6
Cabazitaxel	6
Abiraterone	6
SL-801	1
177Lu-PSMA-617	1
Rucaparib	1
**Prior Systemic Therapy**	
Abiraterone	9
Samarium-153	1
Bicalutamide	16
Cabazitaxel	3
Cyclophosphamide	1
Degarelix	1
Docetaxel	7
Enzalutamide	13
Flutamide	1
Itraconazole	1
Lupron	20
Nilutamide	5
Radium	1
**Administeration of sipuleucel-T**	
1st line (prior ADT only)	11
2nd line	6
≥3rd line	3

177Lu–lutetium-177 radiometal, ADT–androgen deprivation therapy, Beta2–beta-2 microglobulin, CRP–C-reactive protein, ECOG–Eastern Cooperative Oncology Group, Gy–Gray, LDH–lactate dehydrogenase, N–number, PSA–prostate specific antigen, PSMA–prostate-specific membrane antigen, SAbR–stereotactic ablative radiotherapy, STD–standard deviation, SL-801–XPO1 (exportin-1) inhibitor, WBC–white blood count.

**Table 2:** MDSC percentages in PBMCs. (**A**) MDCS sub-populations at baseline in the investigated patients and healthy donors. (**B**) Changes in the MDCS sub-populations in patients grouped based on their clinical response. (**C**) Changes in the MDCS sub-populations in patients grouped based on their PSA biochemical response.

**(A)**			
**Parameter**	**Patients (n = 9)**	**Healthy Donors (n = 3)**	***p* Value**
M-MDSC baseline	9.73 ± 6.68	0.67 ± 0.31	0.004
e-MDSC baseline	0.58 ± 0.71	0.17 ± 0.26	0.362
**(B)**			
**Parameter**	**Responders (n = 5)**	**Non-Responders (n = 4)**	***p* Value**
M-MDSC baseline	12.06 ± 8.16	6.83 ± 3.14	0.269
M-MDSC FU	9.56 ± 4.30	12.05 ± 6.59	0.514
∆M-MDSC	−2.50 ± 7.06	5.23 ± 3.49	0.088
e-MDSC baseline	0.49 ± 0.39	0.69 ± 1.06	0.711
e-MDSC FU	0.45 ± 0.36	0.10 ± 0.09	0.065
∆e-MDSC	−0.04 ± 0.54	−0.59 ± 1.00	0.321
**(C)**			
**Parameter**	**Responders (n = 4)**	**Non-Responders (n = 5)**	***p* Value**
M-MDSC baseline	14.25 ± 7.54	6.12 ± 3.14	0.063
M-MDSC FU	10.33 ± 4.56	10.94 ± 6.23	0.874
∆M-MDSC	−3.93 ± 7.28	4.82 ± 3.16	0.044
e-MDSC baseline	0.55 ± 0.43	0.60 ± 0.94	0.917
e-MDSC FU	0.40 ± 0.39	0.21 ± 0.27	0.420
∆e-MDSC	−0.26 ± 0.53	−0.39 ± 0.97	0.793

M-MDSC—monocytic-myeloid derived suppressor cell; e-MDSC—early stage-MDSC; PBMC—peripheral mononuclear blood cell; ∆—absolute change; FU—follow-up visit.

## Data Availability

Data was submitted to clinicaltrials.gov (NCT01818986).

## Supplementary Materials

The following supporting information can be downloaded at: https://www.mdpi.com/article/10.3390/biomedicines10061419/s1, Figure S1: Flow cytometry gating strategy to identify M-MDSC and e-MDSC in PBMC samples. M-MDSC–monocytic-myeloid derived suppressor cell; e-MDSC–early stage-MDSC; PBMC–peripheral mononuclear blood cell.; Table S1: (A) Clinical Responders vs Non-Responders (B) PSA Responders vs Non-Responders; Table S2: Adverse Events; Table S3: Changes in the titers of immunoglobulins G (IgGs) against PAP, PA2024, and tetanus antigens. A. Changes in the titers in patients grouped based on their clinical response. B. Changes in the titers in patients grouped based on their PSA response.
