# Supplementary material for: Phase II Trial of Sipuleucel-T and Stereotactic Ablative Body Radiation for Patients with Metastatic Castrate-Resistant Prostate Cancer
Source: Biomedicines. 2022 Jun 15;10(6):1419. doi: 10.3390/biomedicines10061419 (PMC9220346; doi:10.3390/biomedicines10061419)
Supplement: Supplementary file 1 [file biomedicines-10-01419-s001.zip › biomedicines-1758405-supplementary.pdf]

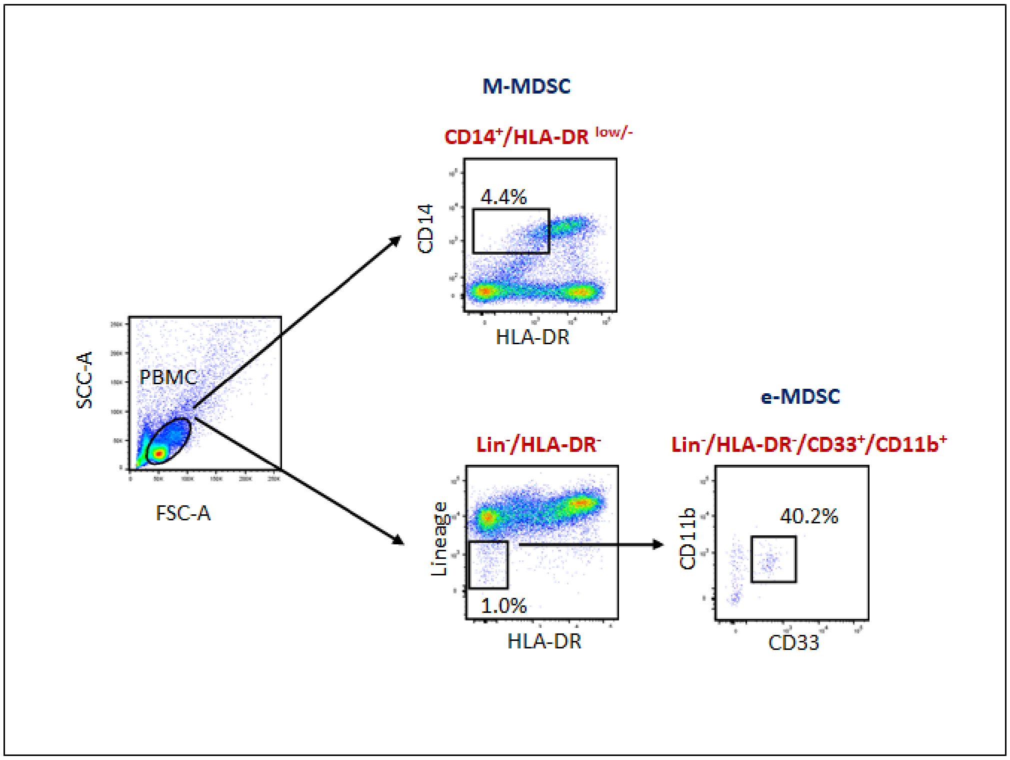

Supplementary Figure S1. Flow cytometry gating strategy to identify M-MDSC and e-MDSC in PBMC samples. M-MDSC–monocytic-myeloid derived suppressor cell; e-MDSC–early stage-MDSC; PBMC–peripheral mononuclear blood cell.

Supplementary Table S1: (**A**) Clinical Responders vs Non-Responders (**B**) PSA Responders vs Non-Responders.

(A) Clinical Responders vs Non-Responders			
Variables	Responder (mean/std)	Non-Responder (mean/std)	p-value
**Age at diagnosis**	63.3/7.6	68.5/5.3	0.32
**Age at enrollment**	71.3/7.9	74.3/6.3	0.59
**PSA value at enrollment (ng/dL)**	12.6/8.2	28.6/39.6	0.52
**Testosterone level (ng/dL)**	7.7/4.7	6.8/4.0	0.78
**LDH (U/L)**	182.8/36.4	209.5/45.9	0.39
**CRP (mg/L)**	10.9/13.2	6.3/1.8	0.61
**Beta2 (mcg/mL)**	2.2/0.4	2.6/0.8	0.35
**Uric Acid (mg/dL)**	4.3/0.9	5.6/0.7	0.05
**WBC (x10^9/L)**	5.7/0.9	5.9/1.1	0.78
Neutrophils (x10^9/L)	3.7/1.0	3.6/1.4	0.95
Lymphocytes (x10^9/L)	1.4/0.4	1.6/0.4	0.5
Monocytes (x10^9/L)	0.5/0.1	0.5/0.1	0.23

Variables	Responder (#)	Non-Responder (#)	p-value
**Race**			1
White, not Hispanic	4	2	
Other	2	2	
**ECOG**			0.2
0	3	4	
1	3	0	
**Grade Group**			0.47
4+	4	4	
3	2	0	
**Original Primary Gleason Score**			1
4+	5	4	
3	1	0	
**Original Secondary Gleason Score**			1
4+	4	3	
3	2	1	
**High Burden Metastatic Disease**			0.57
Yes	3	1	
No	3	3	

(B)PSA Responders vs Non-Responders

Variables	Responder (mean/std)	Non-Responder (mean/std)	p-value
**Age at diagnosis**	62.6/8.1	68.2/4.8	0.27
**Age at enrollment**	72.0/8.4	73.0/6.1	0.85
**PSA value at enrollment (ng/dL)**	8.1/3.24	28.0/35.5	0.43
**Testosterone level (ng/dL)**	7.9/5.1	6.7/3.6	0.7
**LDH (U/L)**	191.8/33.2	195.2/50	0.91
**CRP (mg/L)**	12.1/14.1	6.0/1.6	0.47
**Beta2 (mcg/mL)**	2.3/0.4	2.5/0.8	0.7
**Uric Acid (mg/dL)**	4.1/0.9	5.5/0.7	0.05
**WBC ($x10^9$/L)**	5.9/0.8	5.6/1.1	0.7
Neutrophils ($x10^9$/L)	4.0/0.8	3.4/1.4	0.46
Lymphocytes ($x10^9$/L)	1.3/0.4	1.6/0.4	0.19
Monocytes ($x10^9$/L)	0.5/0.1	0.5/0.1	0.72

Variables	Responder (#)	Non-Responder (#)	p-value
**Race**			1
White, not Hispanic	3	3	
Other	2	2	
**ECOG**			0.17

0	2	5	
1	3	0	
**Grade Group**			
4+	3	5	0.44
3	2	0	
**Original Primary Gleason Score**			1
4+	4	5	
3	1	0	
**Original Secondary Gleason Score**			1
4+	3	4	
3	2	1	
**High Burden Metastatic Disease**			1
Yes	2	2	
No	3	3	

Supplementary Table S2: Adverse Events.

Adverse Event	Grade			Grand Total
	**1**	**2**	**3**	
Alkaline Phosphatase Increased	1			1
Anemia		1		1
Anxiety	2			2
Back pain	1			1
Chills	1	1	1	3
Constipation	1			1
Cough	1			1
Dehydration	1			1
Diarrhea	2			2
Dyspnea	2	1		3
Extrapyramidal Disorder	1			1
Fatigue	10	1	1	11
Fever	2			2

Generalized Muscle Weakness	1			1
Headache	1			1
Hot Flash	1			1
Hypocalcemia	1			1
Hypophosphatemia	1			1
Lethargy	1			1
Lymphocyte Count Decrease	1	1		2
Nausea	5		1	6
Non-Cardiac Chest Pain	1			1
Photosensitivity	1			1
Productive Cough	1			1
Pruritus	1			1
Rectal Pain	1			1
Rectal Urgency	1			1
Thrombolic Event		1		1
Urinary Retention	1			1
Urinary Tract Obstruction		1		1
Urinary Tract Pain		1		1
Urinary Urgency	3			3
Vomiting	2		1	3
White Blood Cells Decreased	2			2
**Totals**	**51**	**8**	**4**	**64**

Supplementary Table S3. Changes in the titers of immunoglobulins G (IgGs) against PAP, PA2024, and tetanus antigens. A. Changes in the titers in patients grouped based on their clinical response. B. Changes in the titers in patients grouped based on their PSA response.

A.

Parameter	Responders (n = 5)	Non-Responders (n = 4)	*p* value
PAP titer baseline	1212.5 ± 1371.4*	662.5 ± 704.0	0.502
PAP titer FU	14,000.0 ± 24,825.8	7700.0 ± 11,988.3	0.664
ΔPAP titer	12,787.5 ± 25,077.5	7037.5 ± 11,854.4	0.693
PA2024 titer baseline	362.5 ± 325.0	225.0 ± 202.07	0.499
PA2024 titer FU	7600.0 ± 12,053.2	4100.0 ± 5827.5	0.620
ΔPA2024 titer	7237.5 ± 12,189.0	3875.0 ± 5930.2	0.638
Tetanus titer baseline	1700.0 ± 1148.9	2700.0 ± 2759.2	0.528
Tetanus titer FU	3000.0 ± 2476.6	4450.0 ± 5700.0	0.657
ΔTetanus titer	1300.0 ± 1437.6	1750.0 ± 3130.0	0.803

B.

Parameter	Responders (n = 4)	Non-Responders (n = 5)	*p* value
PAP titer baseline	550.0 ± 433.01	1170.0 ± 1288.2	0.462
PAP titer FU	17,600.0 ± 29,098.5	6800.0 ± 10,575.4	0.464
ΔPAP titer	17,050.0 ± 28,884.4	5630.0 ± 10,737.8	0.438
PA2024 titer baseline	216.7 ± 175.6	340.0 ± 311.05	0.560
PA2024 titer FU	9866.7 ± 13,678.2	3440.0 ± 5258.1	0.365
ΔPA2024 titer	9650.0 ± 13,709.0	3100.0 ± 5420.2	0.361
Tetanus titer baseline	1733.3 ± 1404.8	2480.0 ± 2439.7	0.651
Tetanus titer FU	3466.7 ± 2809.5	3880.0 ± 5098.2	0.903
ΔTetanus titer	1733.3 ± 1404.8	1400.0 ± 2821.3	0.858

PAP - prostatic acid phosphatase; Δ – absolute change; FU – follow-up visit.

*IgG raw mean fluorescence intensity.
